# The Perceptions of Women About Their High Experience of Using Crack Cocaine

**DOI:** 10.3389/fpsyt.2022.898570

**Published:** 2022-04-29

**Authors:** Saulo G. Tractenberg, Jaluza A. Schneider, Bernardo P. de Mattos, Carla H. M. Bicca, Bruno Kluwe-Schiavon, Thiago G. de Castro, Luísa F. Habigzang, Rodrigo Grassi-Oliveira

**Affiliations:** ^1^Graduate Program in Psychology, School of Health and Life Science, Pontifical Catholic University of Rio Grande Do Sul (PUCRS), Porto Alegre, Brazil; ^2^Decision in Context, Research Center for Psychological Science, University of Lisbon (ULisbon), Lisbon, Portugal; ^3^Department of Psychology, Federal University of Rio Grande Do Sul (UFRGS), Porto Alegre, Brazil; ^4^Translational Neuropsychiatry Unit, Department of Clinical Medicine, Aarhus University, Aarhus, Denmark

**Keywords:** substance use, crack cocaine, sex differences, qualitative study, thematic analyses

## Abstract

**Introduction:**

The aim of this study was to explore the perceptions of women about their experience in using crack cocaine, discussing their motivations for using it and the repercussions in their lives.

**Objective:**

To investigate these experiences, a qualitative exploratory study was conducted, using the inductive thematic analyses of the content.

**Methods:**

Eight female crack cocaine users took part in this study. They were assessed by a semi-structured interview, addressing the crack cocaine use experience. Four main themes emerged in the interviews: (1) crack cocaine “high” experience; (2) symptoms related to crack cocaine use; (3) circumstances of crack cocaine use; and (4) crack cocaine use consequences.

**Results:**

The main perceptions reported by the users were related to a feeling of being disconnected to the world preceded by a pleasant experience, especially during the first moments of use. They revealed that the drug fulfills a key role of coping strategy to handle with negative thoughts, emotions or life experiences. An important influence of social issues was reported in relation to the onset of crack cocaine use. Negative consequences and significant impact on their lives appeared in their reports, regarding the loss of family ties, involvement with prostitution, traumatic experiences and violence.

**Conclusion:**

Taking together all women's perceptions suggests that beyond the positive immediate rewarding effect, the maintenance of use might be related to the dissociative experience and self-medication role, acting as negative reward by relieving of negative life experiences that, in turn, are both cause and consequence of the drug use.

## Introduction

Cocaine Use Disorder (CUD) represents a serious public health problem with about 20 million people using the drug annually worldwide. Cocaine consumption has been increasing over the last decades. Recent reports from Word Drug Report 2021 estimated a potential increase of 11% in global drug use for 2030, with pronounced impact in low- and middle income- countries, which could be, in part, explained by the availability of the drug in derived forms that provide greater returns to the drug market (1). In South American countries, for example, crack cocaine “rocks”– a cocaine base obtained from hydrochloride conversion for smoking usually through a pipe (2)—has been widely consumed, such as in Brazil (3). Despite there is a few studies of epidemiological data on specific increase of crack consumption, Brazilian drugs reports have been estimated that 1.3% of drug users consume crack cocaine form annually (4). Crack cocaine is produced with the same chemical base from cocaine, but with less amount of water, which results in a tropane alkaloid composition. Despite both forms having distinct ways of administration, time of action and half-life duration, they have similar active components, being capable to produce psychostimulant effects (e.g., euphoria, energy gain, increased psychomotor activity and alertness, reduced appetite and sleep needs) in the user (5). At high doses or in a chronic and prolonged use form, otherwise, both cocaine and crack cocaine could trigger negative emotions (e.g., mood and anxiety symptoms), paranoia, impulsive and aggressive behaviors, and physiological reactions (6). Clinical evaluation of crack cocaine users (CCU) has been suggested the presence of more pronounced symptoms and negative psychosocial effects when compared to those who consume the drug in powder form (7, 8) or any other drugs, increasing the demand for CUD treatments.

The CCU profile is not different from other drugs with prevalence rates being higher among men, however, in the last few years, drug reports and few studies have been suggested an increase of crack cocaine consumption among women (1, 9, 10). This new perspective leads to an effort from the Brazilian scientific community to investigate potential sex specificities in a range of target-points, which is in line with an international tendency to explore and integrate on addiction studies sex and gender differences as a main issue for investigation (11–13). Several factors are being highlighted as potential differences between sexes in relation to drug use and addiction, including the psychoactive effects of each drug, the patterns and motivations of use, the dependence and withdrawal symptoms and, finally, the treatment challenges and strategies (14–16).

Despite still incipient, some findings have been suggested that women have more severe pattern of drug use in association with higher rates of psychiatric comorbidities and psychosocial problems (e.g., familiar, work-related, legal, and criminal problems) (17). It might contribute to social stigmatization among women users, influencing the appearance of high-risk behavioral profile in this population (9, 14, 15, 18–20). A review of Brazilian crack cocaine studies, for example, reinforced such idea indicating that drug consumption among women increase their exposure to vulnerability regarding specific sex issues, including gestation health problems, intrapersonal and domestic violence, prostitution and moral judgment (21). The motivations that lead women to seek the drug is also suggested to be different when compared to men. Evidence from studies with CCU women revealed that there is a lot of emotional drive involved in motivation for drug seeking-behavior, especially related to the attenuation negative emotions. In contrast, men are generally motivated to drug consumption for more positive reinforcement reasons, such as the pleasure experience and reward-related effects (22, 23).

In this sense, it seems that different trajectories lead to crack cocaine use and progression to addiction among women and men. This could be influence by a range of factors that produce distinct experiences associated with the drug (11), opening an interesting field for both quantitative and qualitative investigations. Qualitative studies allow us to explore and deeply comprehend the personal experiences, motivations, and thoughts of the users about their own addictive condition. Investigating these meanings might represent an important contribution for individual and community interventions and, at final step, public health policies, since highlight subjective personal experiences (e.g., motivations and repercussions in life) and needs that could be shared by addicted individuals. Also, exploring women's perceptions contribute to improve the knowledge of specific sex factors underlying crack cocaine addiction that are still poorly described and understood. For this reason, the current study aimed to explore the perceptions of women about their experience in using crack cocaine, addressing their own motivations for use and the repercussions in their lives. For this purpose, a qualitative exploratory design was conducted using the inductive thematic analyses of the content.

## Methods

### Participants

Eight female crack cocaine users, who were admitted into a detoxification unit for alcohol and other drugs in the city of Porto Alegre, Rio Grande do Sul, participated in this study. The inclusion criteria were (a) voluntary hospitalization; (b) crack cocaine as the main drug of use, and; (c) diagnosis of Crack Cocaine Use Disorder (according to the DSM-5 criteria) and prior assessed by SCID-V interview. The exclusion criteria were as follows: the presence of psychotic symptoms; psychomotor agitation and/or disorientation; incapability of understanding and sustaining a conversation; and presence of chronic diseases (e.g., HIV or metabolic diseases). These exclusion criteria were not limited to this study specifically, being applied by our larger cohort from which this qualitative study derives. All participants remained during the 21-day period of detoxification with no access to alcohol, tobacco, or any other drug, as prescribed by the mental health unit.

Participants had a mean age of 29.2 years (SD = ±8.3). On average, the number of years of education was 8.2 years (SD = ±2.5) and most of them (*n* = 6) were unemployed. Only one participant was married and the average number of children among them was 1.1 (SD = ±1.31). Participants reported a total number of 5.3 (SD = ±2.7) previous hospitalizations for treating substance related disorders. Most of them were polysubstance users, using tobacco (*n* = 7), alcohol (*n* = 6), marijuana (*n* = 5), and cocaine (*n* = 3) in addition to crack cocaine. The mean age of crack cocaine onset use was 22.1 years (SD = ±8.5).

### Ethical and Data Collection Procedures

All participants were invited and provided informed consent to participate in the study. The Informed Consent Form was presented, and the aims of the study were explained to the participants before the interviews took place. The interviews were individually performed by trained psychologists in a private room inside the unit. All interviews were conducted in a single session and lasted ~1 h. This study was approved by the Ethics and Research Committee of the institutions involved.

Data were collected using a semi-structured interview, which aimed to explore and comprehend the crack cocaine consumption experiences of these women. Interviews started with two leading questions, “*How would you describe being high on crack?*” and “*How was the experience of using it? /How did you feel when you used it*?” Based on the narrative described by the participants, additional questions could be formulated. Consequently, the following topics were also addressed: physiological, cognitive and emotional effects caused by the drug use; comparison of the effects of crack cocaine use with those of other drugs consumed; and comparison of current and initial effects of crack cocaine use. All interviews were recorded in audio format and transcribed for later analysis. The leading and additional questions are presented in Table 1.

**Table 1:** Guiding questions of the interview.

**Leading questions**
*“How would you describe being high on crack?”*
*“How was the experience of using it?/How did you feel when you used it?”*
**Additional questions**
*“Is there any difference between your current use experience and the first time you used it?”*
*“How you describe the differences between crack and other drugs (e.g., cocaine, alcohol, marijuana) usage?”*
*“How would you describe your experience after the crack high ends?”*
*“For how long do you feel the effects of the crack‘s high?”* *“Could you tell us in which moments do you usually use crack”*

### Data Analysis Procedures

Inductive thematic analyses were conducted based on the proposal by Braun and Clarke (24) in six steps. In Step 1 (data collection and recording), the recorded interviews were transcribed by two research assistants. In Step 2, a free reading of the transcribed text was performed, and, in the sequence, preliminary codes were created from the relevant data according to the study objectives. In Step 3, the coded data was sorted out by clustering extracts according to potential themes. In Step 4, the themes were reviewed, with the content of the data and consistency within emergent themes and subthemes being observed. At this stage, two independent judges, with expertise on substance use disorders, reviewed the themes and subthemes, considering the data and the content of excerpts from the analyzed interviews. Both analyses were compared to verify possible discrepancies in relation to themes and subthemes, and these disagreements were discussed until a consensus was reached. In Step 5, themes and subthemes were named and finalized, with examples of consistent and representative extracts. Finally, in Step 6, the results were formally written and interpreted considering the literature background (24, 25).

## Results

**Figure 1:**
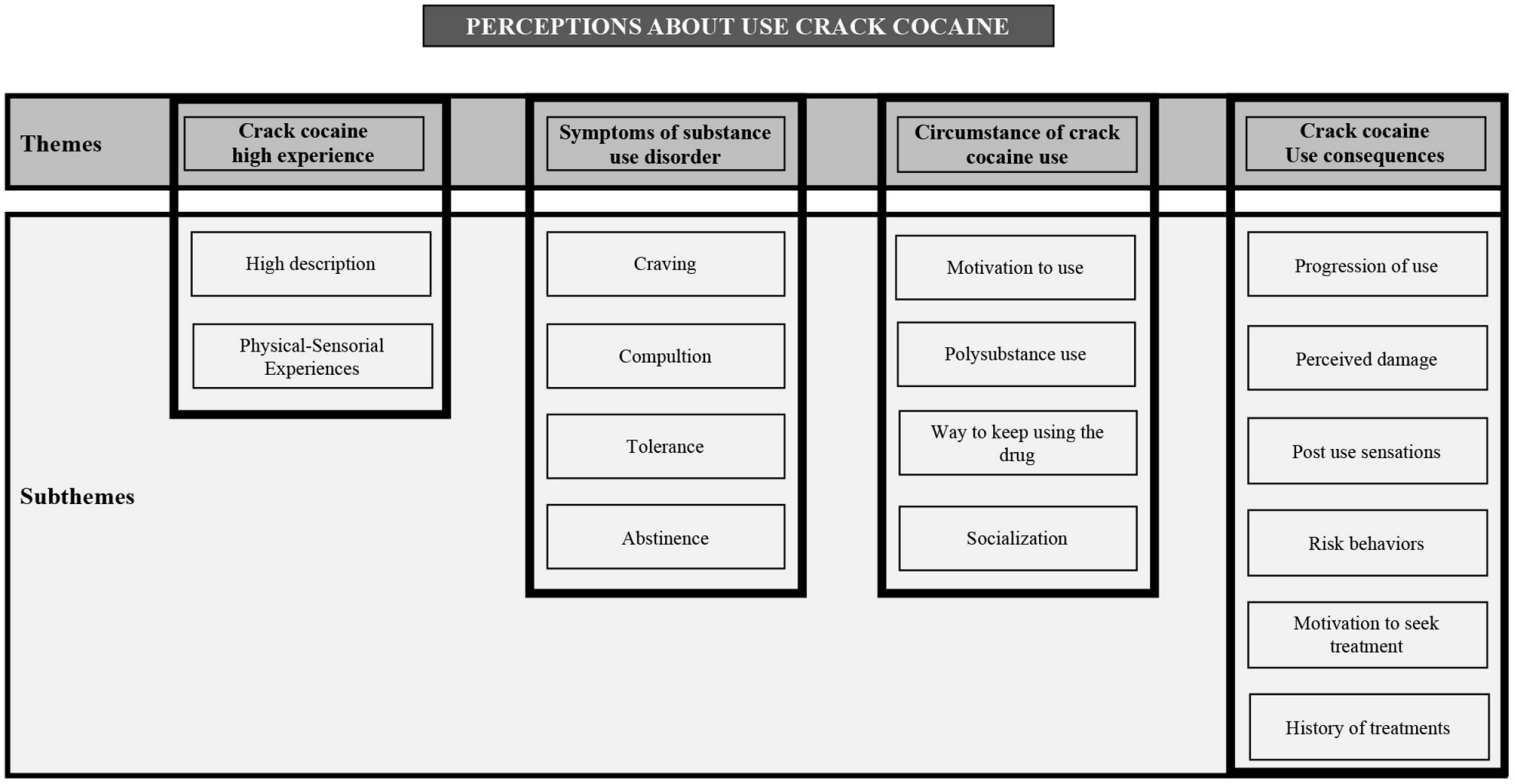
Thematic analysis—themes and subthemes.

Based on the thematic analysis, four main themes emerged: (1) Crack cocaine high experience; (2) Symptoms of substance use disorder; (3) Circumstances of crack cocaine use; and (4) Crack cocaine use consequences. In each theme, specific subthemes were identified as shown in Figure 1.

The theme “Crack cocaine high experience” was related to the leading question of the interviews. This theme was divided into two subthemes: (1.1) “Description of the high,” which demonstrated a pleasant experience, especially during the first moments of crack cocaine use, represented by words such as: ‘*ecstasy’*, ‘*euphoria’*, ‘*horny’*, ‘*accelerate’*, ‘*well-being*’, ‘*relax’*, ‘*relief’* ‘*soothe’*; and (1.2) “Physical-Sensorial Experiences” including aspects described by the users about their experiences while under the effect of the substance, such as sensations of ‘*peace*’, ‘*thinking of nothing’*, ‘*sped up*’, paranoia, hallucinations and physical symptoms such as heart beating fast and panting.

The second theme, “Symptoms of substance use disorder,” was divided into four subthemes: (2.1) “Craving,” which they described as an uncontrollable desire to use crack cocaine, with physical sensations (‘*shaking’* and ‘*sweat’*), constant thoughts about the need for intoxication again and about plans for it; (2.2) “Compulsion” revealed the binge use of crack cocaine, such as “*When I start, I don't want to stop anymore*,” “*I don't know how to use just a little bit of drug*” or “*You spend the whole day busy* (in order not to use it)”; (2.3) “Tolerance” indicating the attenuation of crack cocaine effects; and (2.4) “Abstinence” symptoms, revealing the symptoms that users felt due to the absence of the drug.

Three subthemes emerged from the third theme “Circumstance of crack cocaine use,” which addresses the context and forms of consumption: (3.1) “Motivation to use,” was related to positive expectations toward the crack cocaine use effects, especially on the attenuation of negative feelings; (3.2) “Polysubstance use,” revealed the consumption of other drugs during the lifespan of the participant; (3.3) “Ways to keep using the drug,” identified how users maintain their consumption, such as selling personal belongings and, most notably, attempting sex work and prostitution in exchange for or to obtain money to purchase the substance, and (3.4) “Socialization,” which revealed the social experiences during the participants' crack cocaine consumption.

The fourth theme “Crack cocaine use consequences” was divided into six subthemes: (4.1) “Progression of use,” addressing the main aspects for the onset and progression to crack cocaine dependence; (4.2) “Perceived damages,” describing the negative consequences of the consumption of crack cocaine identified by the users themselves; (4.3) “Post use sensations,” considering the negative aspects when ceasing the intoxication; (4.4) “Risk behaviors,” which identified risk situations where participants engaged on to obtain or to use crack cocaine, and, finally, (4.5) “Motivation to seek treatment” and (4.6) “Treatment history,” revealing the participants' description of their relapses and history of treatments attempts (Table 2).

**Table 2:** Themes, subthemes, and representative thematic units.

**Themes**	**Subthemes**	**Representative thematic unit**
1. Crack high experience	1.1 High description	[...]To get out from world, to get out from earth, to get out from me. To not have to think, sons or anything else, just... just get out, you know?
	1.2 Physical-Sensorial Experiences	[…]I feel excited, I get up, I start to walking around and daydreaming. I daydream about people... that them are staring at me, looking at me different... I want to hit them, react, hit this way (move both hands as is hitting something)... so I already want to move forward, I want to move forward to fight, I want to hit, you know?
2. Substance use disorder symptoms	2.1 Craving	[...]I know it's stronger than me, my hands are sweating, I‘m shaking, I’m going to step on the house, I’m going shake again I’m sweating and I put it into my head that I want it, I want it. I can’t stop thinking about it. I‘m craving for it and it already gives me a stomachache, headache, body pain...
	2.2 Compulsion	[...]Actually I’m pretty compulsive, so, regarding the drug. The more I use, more I want to use, understood? [...] There is no ending, differently from those people who stop. When I start, I do not want to stop.
	2.3 Tolerance	[...]Was... And I was already... the drug didn’t have more effect because I was using for many days, almost two weeks. Smoking repeatedly and smoking. So I can’t feel more the effects.
	2.4 Withdrawal	[...]Now, now, I’m starting to feel better it’s complete, because I had nightmares all night long. All nights, so I dream that I’m smoking. It’s awful.
3. Circumstance of crack use	3.1 Motivation to use	[...]Desire... desire. Firstly, the addiction. The addiction, the desire of use again and again and again... Feel the high. To be on my trip again. To not feel any guilty.
	3.2 Polysubstance use	[...]Oh, I don’t even know how to explain it to you, but it’s... more powerful than cocaine. Cocaine you aspirate a little and you feel well... your conversation goes and goes and goes. The crack already punches like that, you know? [...] I’m not paranoid about picking, but I get more aggressive when I’m drinking [...]
	3.3 Ways to keep using the drug	[...]I wanted to smoke. So I went to my friend’s garage, he’s a mechanic and asked for twenty reais. He wants to have sex with me, so I had sex with him, right? I got my twenty reais and went away. I don’t know... okay, I got him, I got twenty reais and went to buy drugs. I got ten and I smoked it. I got more ten and also smoked it. After finished, I was like this, right? No money. Then I had sex with... I kind of... drug dealer to get more crack stones, so he gave me more.
	3.4 Socialization	[...] There are some that enjoy smoking alone, some times I end up smoking alone, but I do one or two times and I have to go near my friends. Just one or two alone, like a selfish.
4. Crack use consequences	4.1 Progression of use	[...]Since that day, the first time that I use, I started to smoke every weekend, like a couple of crack stones. After, there is a time that I started to disappear, all night long and I lost completely the control of that... Until I ended up in the hospital.
	4.2 Perceived damage	[...] I came here very sad, I tried to kill myself. [...] Crack no, crack is a prison. You smoke one and get locked.
	4.3 Post use sensations	[...]When you stop, you’re ashamed of the things you did. You look at yourself in the mirror, your face are all dirty, gray... it is a great humiliation. [...]All the time. Then I start to speak, my breath returns to normal, I start to look to the people normally and that’s how it is.
	4.4 Risk behaviors	[...]I stabbed myself, right? In those five minutes that I‘m out of me because the crack high... I stabbed myself in the foot and took six stitches. In addition to stab me, I still put my finger inside my wound and started to move, like this, and did not feel anything, nothing, no pain... [...]I... I’m with a person that I like, but I do not feel love. So I have sex to him because I can get crack. So I have a relationship with him because of it. Understood? Because he helps me, because he gives me crack. You know? But he helps me at home too. Because I‘m working too in the streets to get crack, I have sex with others to maintain my addiction, got it?
	4.5 Motivation to seek treatment	[…]I lost my father, it’s been 11 months since he died and he was an alcohol addict. [...]And he said to me, ’daughter, look, you’re between life and death, you’re going to stop... I hope you stop before. And what happened to me, I’m going to stop, I stopped. This is why I‘m here. “[...]I’ve seen five people being killed and this motivate me to stop the drug use. Then I started to want to be hospitalized. Because there, outside, the death passed nearby and not take me.
	4.6 History of treatments	[...]After that I started to go to drug-treatments, being hospitalized... I have consecutive hospitalizations and some short time relapses... then hospitalized again, relapsed, hospitalized again and I stayed until I’m able.

## Discussion

The present study aimed to explore the perceptions of women regarding crack cocaine use and their thoughts about the representative role of it in their lives, including the motivations to use the drug and the consequences of it. The user’s speech suggested that one of the main perceptions related to the crack cocaine use is a pleasant experience, especially during the first moments of use, in addition to a feeling of being disconnect to the world. Also, crack cocaine users revealed that the drug fulfills a key role as a coping strategy to handle with negative thoughts, emotions and/or life experiences. Crack cocaine was considered a drug with distinct characteristics compared to other drugs, including cocaine itself. The main differences pointed by the users were related to intensity and rapid effects. Furthermore, users suggested that the onset of their use was influenced by interpersonal relationships, highlighting the progression of use as fast and uncontrolled. The risk exposure associated with both use and continued use are recognized, as exemplified by putting themselves on high-risk situations, joining sex work and family detachment.

The description of the experience of crack cocaine intoxication was characterized by intense pleasure and euphoria: “*I don’t know, I think it is this, I get really agitated… and euphoric*,” sometimes followed by an immediate suffering relief: “*no pain, no pain whatsoever, nothing at all. After the effect ceases, everything – the pain comes back, it comes…*” The drug relief effect is consistent with reports indicating that CCU women commonly deal with their negative emotional states when using drugs: “*And then I fall to the ground, and then I come… feel, I start to feel pain, start to feel everything*.” (User 3). Using crack cocaine was perceived as the only alternative to deal with problems, as exemplified in the following statements by one of the participants on the effects of crack cocaine: “*if I feel some pain, if I have something, it’s all gone at once*.” And “*get out of the world, get out from earth, get out from myself* . *Not think about anything, not think about son, nor on problem, nor on mom, nothing at all, so, it’s just… just get out of myself, you know?*” (User 4 and 5). According to Cafure (26), the fast-acting mechanisms of the drug in addition to its reinforcement properties, including the pleasure experience (positive reinforcement) and/or the attenuation of emotional suffering (negative reinforcement), which could be understood as a maladaptive coping strategy allowing the reinforcement of the drug seeking behavior (27).

It is distinct to what is referred by men users, who usually tend to use the substance seeking pleasure (22, 23). There are discussions pointing out that, especially among women, the drug use could be characterized as a self-medication role to handle with negative feelings and life experiences, such as early childhood or posterior experiences. Self-medication hypothesis suggests that individuals attempt to mitigate and cope with negative symptoms induced by different psychological conditions, including Depression, Posttraumatic Stress Disorder (PTSD), and drug addiction (28–32). In this sense, the effects of crack cocaine were generally described as having a role related to the relief of different sensations considered unpleasant, such as tiredness, guilt, and day-to-day concerns. The drug was depicted as a “*shelter from their problems*.” The participants’ speech related to “*thinking about nothing*” suggests the necessity for escape from problems and concerns and the lack of emotional adaptive coping skills. Some studies already evaluated that women are more likely to sustain beliefs related to not being able to deal with intense emotions, using drugs as an emotional regulation alternative (33–36).

Crack cocaine fits well in this regard since the users reported an intense and fast drug effect. However, these effects tend to present a short-time duration, followed by a strong desire to administer and experiencing it again, corroborating the indicatives of high levels of craving and withdrawal symptoms, as well as a drug seeking and compulsive behavior (37, 38). It reflects what one user described as a compulsive use “*I used it fifteen days straight. Not sleeping, no food, no nothing. Fifteen days. I received from my workload, received my salary and ended up smoking. Stayed at home, locked in my house, smoking, smoking, smoking, smoking*” (User 1). This pattern of crack use usually is observed in “cracklands” along different Brazilian cities, where users meet in larger open spaces to use crack reflecting vulnerable conditions and extreme poverty (39). Such compulsive behavior and way of life led to several issues, especially those related to their relatives. Despite having awareness about it and the adverse life consequences, the CCU women reported that they feel unable to control their addictive behavior, as referred in the participants’ speech: “*When I start, I don’t want to stop anymore*”, “*I don’t know how to use just a little bit of drug*,” or “*you spend the whole day busy* (using it).” A study from Freitas et al. (40) with CCU men, for example, revealed that they also have their own perception regarding the negative consequences and that this perception was not capable to influence the cessation of drug use.

The perceived negative impact of crack cocaine use mentioned before, however, was not referred in the same degree in relation to risk-behavior perception. Risk perception seem to be reduced and/or distorted among drug users (41). Reports about hurting oneself such as: “*I already hurt myself with a knife, fooling around with a knife. I stabbed my foot, I had six stitches”* (User 2); “*I worked as a hooker, I had my clients. So, I would do what they asked. I did not care; I didn’t feel pain*” (User 6); were examples of exposure to high-risk situations that seems to be dissociated from potential negative consequences. In both described speeches, the previous awareness of negative consequences is absent and corroborating to risk-behavior exposure.

The craving symptoms experience described by some participants indicated that such symptoms are enhanced following the pain relief experience, inducing the search and desire to experience the initial sensation of the crack cocaine high again. In CCU men, for example, it was reported that expectations about changes in craving sensations and negative feelings after crack cocaine high cessation are important for the maintenance of the crack cocaine consumption behavior (40). CCU women findings suggested that crack cocaine use could be viewed as a behavior that is chosen for its reinforcing consequences. Thus, the attenuation of undesired craving symptoms was considered an additional reinforcement factor (34, 42). Specifically, our study participants demonstrated in their speeches that they also had to deal with other unpleasant symptoms, including insomnia and nightmares, suggesting that in addition to craving, withdrawal symptoms could also influence their addictive behavior pattern.

Polysubstance use history was another characteristic reported in our sample. There are reports suggesting high prevalence of polysubstance use, such as alcohol and cannabis, among CCU, inducing higher problems with psychiatric symptoms and impulse control (43, 44). Indeed, both substances were the most referred beyond crack cocaine and were perceived as able to induce additional experiences in crack cocaine high. Alcohol use, for example, despite reported as able to reduce the craving symptom experience, was followed by an increase in aggressiveness and violent behaviors. Cannabis use, which was described as capable to induce relaxation and sleepiness: “*The weed has it. Completely. You get that weed, you smoke that little weed, it gives you the couchlock, it gives you a feeling of pleasure, of relief. Sleepiness and dry mouth. And… gets you hungry, right? Weed is this. Weed is a sedative*.” (User 4), in combination with crack cocaine was suggested to induce sociability during the usage. The history of drug use and the differences in high experience between smoked crack cocaine and snorted cocaine also should be pointed: “*The cocaine, happened. The cocaine the effect is normal, it’s… you don’t get out of that thing there, you don’t stay on that anguish “I want more, I want more.” I was satisfied with twenty bucks”* (User 7), referring that the last form tends to be “*weaker*” than crack cocaine, leading to “*less addiction*,” which makes sense due to the quicker absorption of the drug and a more intense reinforcing effect. Cocaine was also mentioned as related to increases in socialization during the use, which is reported rarely when crack cocaine was used alone.

The participants along the interviews frequently pointed out the influence of the social context as one of the main reasons for starting their drug use. Relatives and friends who were cited as the most influences. Some findings already discussed that crack cocaine use among women were commonly influenced by close people (15, 21, 45). Curiosity and attempt to enhance sociability, for example, were reported by women as one of the main motivations to the onset of use. Most of the users revealed similar history of crack cocaine use progression, in which use was sporadic at the beginning and quickly became daily and compulsive. The search for socialization was also suggested to be related to the maintenance of use. Estimates have suggested about 80% of Brazilian CCU choose to use the drug in openly in public spaces of social interaction (4, 39, 46). Interestingly, the users reported that they avoid using crack completely alone, which is different than what has been observed among CCU males (47). According to participants, even without active communication, the presence of other users has a protective effect during the consumption. Higher social vulnerability experienced (e.g., traumatic and violence experiences) by CCU women when compared to men could influence the search for protection during consumption (48). Moraes et al. (21) emphasizes that the sociability of women during the use of crack cocaine should be explored by future studies and can be considered as an important sex difference in crack cocaine use.

The vulnerable condition of CCU women has been discussed by some studies, indicating that this population has a high-risk profile for traumatic exposure, as well as to sexually transmitted infections (STIs), since they often engage in sex trade in order to obtain money to maintain drug consumption (49–52). CCU women that often exchange sex for money or drugs were suggested to be three times more likely to develop syphilis as well as to be victim of violence when compared to men (14). Crack cocaine use was also identified as a risk factor for trauma related disorders. Data from a previous study found that CCU women have high rates of exposure to traumatic events, with more than 80% reported having experienced or witnessed an actual or risk situation of sexual or severe aggression (53), which corroborates the evidence suggesting that women who are CCU were more likely to report lower education, childhood maltreatment and unstable housing situation (53). Furthermore, the lack of physical care, such as not eating, not sleeping and not performing adequate hygiene was perceived as additional damage of the crack cocaine use, increasing the susceptibility for health and mental health concerns (54).

Regarding the aspects related to treatment seeking, family inquiry appeared as one of the main reasons for CCU women asking for help. Some participants, in their speech, demonstrated that the possibility of taking care of their children again or the reunification is the major motivation for engaging in substance use disorder treatment (15, 55). On the other hand, lack of family support, absence of children or the possibility of losing her own child have been identified as potential risk factors for relapse and a barrier to treatment enroll (56). Among the cited reasons by women with children reported in a previous study (57), the fearful of losing custody of their child was associated with less likely to enter in treatment programs. Women with CCU are known as a vulnerable group with complex unmet needs, for this reason, the risk of losing child care could represent additional challenge for substance use treatment. Interestingly, almost of half of women (who are mothers) receiving treatment already had experienced the least of care at least one time in their lives (58). In this perspective, the participants highlighted that one of most perceived negative consequence of crack cocaine use was the loss of family, especially for those who are mothers. In the review study of Doab (58), it was discussed that keeping the mother with their children could improve rates of treatment adherence, since women spend more time enrolled in drug treatments. Thus, it can be suggested that family and social detachment might aggravate the vulnerability condition of these women and consequently increase the risk to crack cocaine use, relapse during detoxification processes or dropout health care treatment programs for substance use (59, 60).

## Conclusion

This study revealed that the crack cocaine high is a very personal experience, being associated with a pleasant feeling, at the intoxication moment. The progression to continued use and, consequently, addiction might represent a coping strategy to attenuate trauma and negative emotions experienced throughout life. CCU onset was commonly influenced by relatives and/or close social relationships (61) and that socialization is part of their addictive behavior. In comparison to the powder form, crack cocaine was perceived as stronger and associated with more negative outcomes, including family and social support abandonment, sex trade involvement and exposure to violence traumatic experiences. Despite our findings contributing to better understand the subjective experience of CCU, it should be interpreted with some limitation and not generalized to larger groups of users. Our study was based on the analysis of the perception from a small sample of CCU women, valuing their own meaning and interpretation of the experience. The sample itself was unique and, for this reason, explore such subjectivity could open questions and potential targets for more directed intervention (e.g., focusing on emotional regulation, traumatic experience and adaptative coping, understanding differences in motivation and drug use trajectory). Additionally, social and/or familial support should be addressed considering its role for prevent relapse and rehospitalization.

## Data Availability Statement

The raw data supporting the conclusions of this article will be made available by the authors, without undue reservation.

## Ethics Statement

The studies involving human participants were reviewed and approved by Ethics and Research Committee of PUCRS. The patients/participants provided their written informed consent to participate in this study.

## Author Contributions

ST contributed with conceptualization, methodology, investigation, analysis, and writing of original draft and reviewed draft. JS contributed with methodology, investigation, and writing of original draft. BM contributed with analysis and writing of reviewed draft. CB contributed with investigation and analysis. BK-S contributed with conceptualization, methodology, investigation, analysis, and writing reviewed draft. TC contributed with conceptualization, supervision of methodology, and analysis. LH contributed with conceptualization, supervision, and writing reviewed draft and RGO contributed with conceptualization, supervision and project administration, and reviewing all writing steps. All authors read, and approved the submitted version.

## Funding

This study was supported by NIDA and the Fogarty Foundation (R01DA044859) and Conselho Nacional de Desenvolvimento Científico e Tecnológico (CNPq) (Grant No. 466802/2014-5). The funding sources had no involvement in study design; in the collection, analysis, and interpretation of data; in the writing of the manuscript; and in the decision to submit the manuscript for publication.

## Conflict of Interest

The authors declare that the research was conducted in the absence of any commercial or financial relationships that could be construed as a potential conflict of interest.

## Publisher's Note

All claims expressed in this article are solely those of the authors and do not necessarily represent those of their affiliated organizations, or those of the publisher, the editors and the reviewers. Any product that may be evaluated in this article, or claim that may be made by its manufacturer, is not guaranteed or endorsed by the publisher.
